# DENIS: Solving cardiac electrophysiological simulations with volunteer computing

**DOI:** 10.1371/journal.pone.0205568

**Published:** 2018-10-16

**Authors:** Violeta Monasterio, Joel Castro-Mur, Jesús Carro

**Affiliations:** Universidad San Jorge, Villanueva de Gállego, Zaragoza, Spain; Universiteit Gent, BELGIUM

## Abstract

Cardiac electrophysiological simulations are computationally intensive tasks. The growing complexity of cardiac models, together with the increasing use of large ensembles of models (known as populations of models), make extensive simulation studies unfeasible for regular stand-alone computers. To address this problem, we developed DENIS, a cardiac electrophysiology simulator based on the volunteer computing paradigm. We evaluated the performance of DENIS by testing the effect of simulation length, task deadline, and batch size, on the time to complete a batch of simulations. In the experiments, the time to complete a batch of simulations did not increase with simulation length, and had little dependence on batch size. In a test case involving the generation of a population of models, DENIS was able to reduce the simulation time from years to a few days when compared to a stand-alone computer. Such capacity makes it possible to undertake large cardiac simulation projects without the need for high performance computing infrastructure.

## Introduction

**Fig 1:**
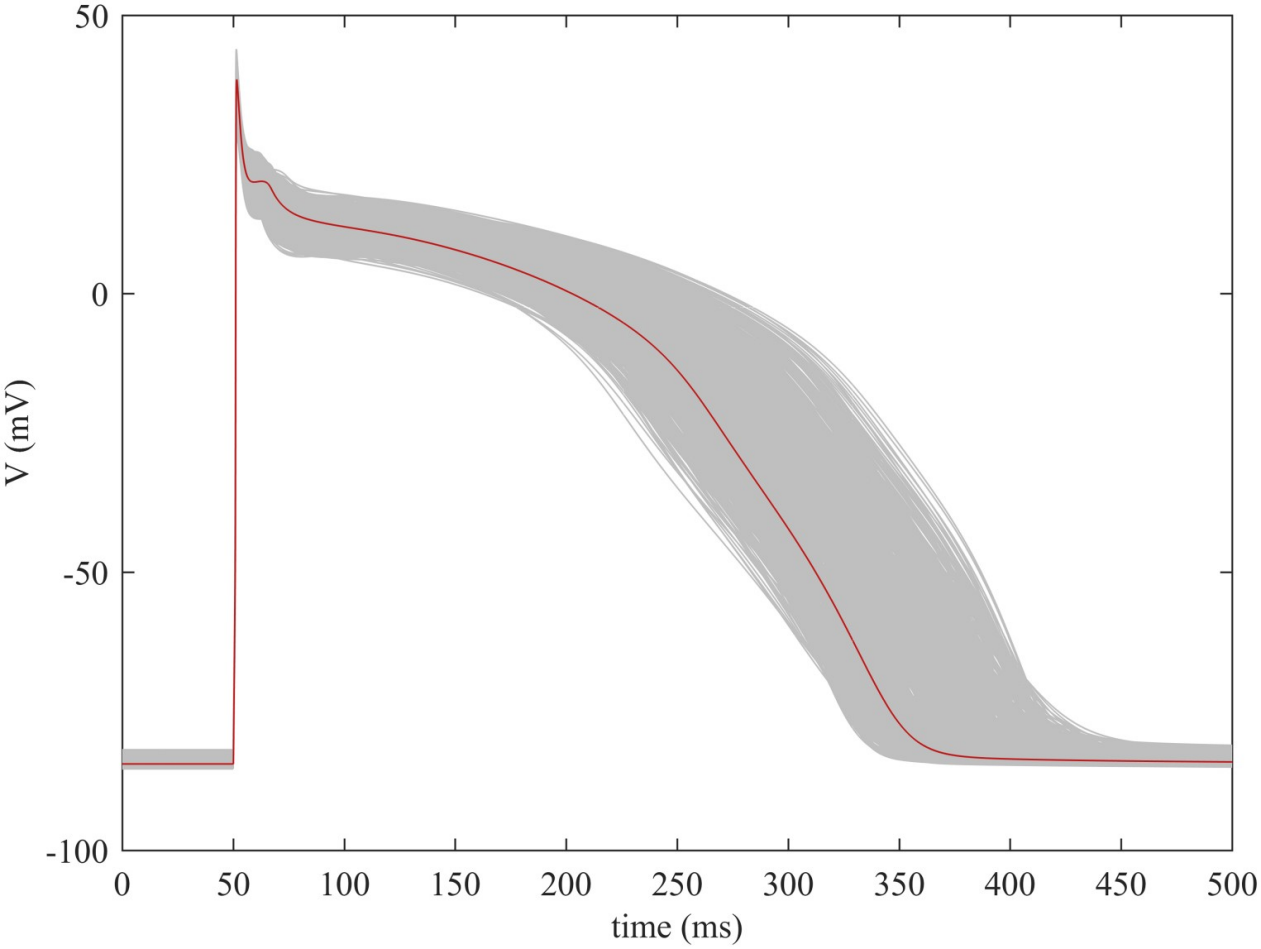
Ventricular action potentials generated with the Carro *et al*. model [1]. The thick red line represents the default output of the model. The grey lines represent 1000 variations of the model, generated by modifying 12 parameters.

Mathematical models of the heart’s electrical activity are a valuable tool for improving our understanding of cardiac electrophysiology. In particular, models of the electrical processes in cardiac myocytes can be used to understand the cells’ functioning under normal conditions, or under alterations such as those produced by diseases or drugs. Fig 1 depicts an example of the typical voltage variation across the cell membrane of a ventricular myocyte (thick line), known as the *action potential* (AP), simulated with the Carro *et al*. model [1].

Over the years, the complexity of cardiac electrophysiological models has increased considerably. For example, the pioneer model by Noble (1962) [2] contained three electrical currents and four state variables, while a recent model like Carro *et al*. (2011) [1] contains 14 currents and 39 state variables. Consequently, the computational cost of cardiac electrophysiological simulations has grown substantially.

Electrophysiological models are generally validated by predicting the results of live-cell experiments. However, such experiments exhibit a great deal of variability between cells and between subjects, while most current cell models only represent an “average” action potential. Instead of using just a single model, a current trend is to create a population of models, that is, an ensemble of models with different values of underlying parameters, with the purpose of reflecting such variability [3] (see Fig 1). A recent work following this approach is the study by Gemmel *et al*. [4], where two populations of over 15000 models were generated in order to investigate the sources of variability in cellular repolarization.

Both factors, the growth in model complexity and the use of model populations, make *in silico* cardiac research a computationally expensive endeavour, which can become too demanding for regular stand-alone computers. Supercomputers or computer clusters can be employed to solve such problems. Cardiac electrophysiological modelling tools that can run in multi-core CPUs, GPUs and in high performance computing (HPC) infrastructures include CARP [5], EMOS [6], Chaste [7], TOR [8] and Myokit [9]. Unfortunately, the economic cost of HPC alternatives may not always be affordable for research projects.

In a previous work [10] we followed a different approach: to use Volunteer Computing (VC) in order to gain access to vast amounts of computing power at a very low cost. We presented the DENIS project (Distributed computing, Electrophysiological models, Networking collaboration, In silico research, Sharing knowledge) [11], which is based on the *Berkeley Open Infrastructure for Network Computing* (BOINC) platform [12]. The VC paradigm has been used in computational biology projects such as Rosetta@home [13], RNA World [14] or GPUGrid.net [15]. However, to our best knowledge, DENIS represents the first application of VC to electrophysiological simulations.

The performance of BOINC projects cannot be predicted, but needs to be analysed empirically, due to the high levels of volatility, heterogeneity, and error rates in volunteer computing [16, 17]. In our previous work we described the DENIS architecture and illustrated it with an example of use. In the present work we extend [10] by analysing three major factors that affect the performance of DENIS, with the objective of finding under what conditions it could be most advantageous to use VC for the simulation of cardiac electrophysiological models. In particular, we evaluate the effect of the length of the simulations, the deadline for the tasks, and the total number of simulations to be solved, on the completion time of simulation batches. We also present a test-case simulation of a typical challenge in computational modelling, the creation of a population of models, and we finally compare the performance obtained with DENIS to that obtained with a regular stand-alone computer. Based on our results, we provide recommendations on how to use DENIS to solve massive amounts of simulations accurately—that is, with a numerical outcome equal to that obtained by a stand-alone computer—within an acceptable time, since the ultimate goal of our work is to make DENIS freely available and useful for the cardiac modelling community.

## Background

DENIS uses the resources of a network of volunteers to perform computational operations requiring a huge quantity of computing power. The problems to be solved by DENIS are divided into small parts, and each part is sent to a volunteer’s computer (a *host*) to be carried out. The reader is referred to our previous work [10] for a full description of the DENIS architecture. The remainder of this section summarizes the details necessary to understand the experiments described in the following sections.

DENIS follows a client/server architecture. Volunteers need to install the BOINC client to join the project. Once the BOINC client is installed, volunteers must select DENIS from the list of BOINC projects provided by the client. The client then downloads and runs the DENIS Simulator, which is an application that includes the electrophysiological models and a mathematical solver. The current version of the DENIS Simulator includes 12 cardiac models and can be expanded by importing models described in the CellML language [18].

Scientists send groups of simulations (*simulation batches*) to the DENIS server using a cloud service. The server stores the scientists’ input data files, creates the corresponding tasks and sends them to volunteers. Volunteers complete the tasks with the DENIS simulator and send the results to the server. In the server, results are validated and sent back to the scientists’ cloud. If a result is deemed correct and arrives before a predefined deadline, the volunteer receives credit. The BOINC credit is just a measure of how much work a volunteer host has completed and has no monetary value, but it nevertheless represents an incentive to volunteers, who may even compete for it [19]. For the DENIS project, one unit of credit represents 1/200 day of CPU time on a reference computer that does 1,000 MFLOPS based on the Whetstone benchmark [20].

**Fig 2:**
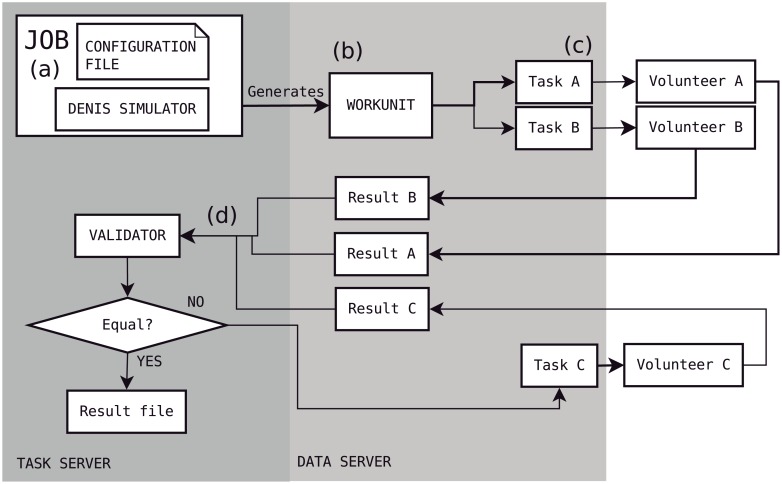
Workflow of a simulation.

Fig 2 details the steps needed to complete a simulation. Simulations start with the scientist filling an XML configuration file with the parameters necessary to run a simulation: the model to be simulated, the duration of the simulation, the type of results to be stored in the output file, and others. Each job consists of a configuration file and the DENIS Simulator (Fig 2(a)). When a job is launched it generates a work unit (WU) (Fig 2(b)) that creates two tasks with the same configuration file (Fig 2(c)). These identical tasks are sent to two different volunteers.

Volunteers send back their results, which are compared in the *validator* (Fig 2(d)). This step is essential to ensure that the numerical outcome of simulations does not contain computation errors by one of the hosts, or just random data sent to earn credit. Cross-platform numerical inconsistencies, however, may not invalidate results if they are small enough. In the *validator*, the results of the different volunteers are compared by checking the percentage of error between the two files for each computed value. If all the errors are smaller than 0.1%, the file is marked as valid, otherwise they are considered different.

If the outcomes from the two tasks are not equal, an additional identical task is generated and sent to another volunteer. An additional task will also be generated if one of the first tasks misses the deadline, defined as the maximum allotted time between the reception of the task by the host and the reception of results. The process of creating an additional task is repeated until at least two tasks produce the same results timely, with a total limit of 10 additional tasks. If that limit is reached without getting a successful result, the simulation is marked as “not valid” and reported in a log file, so that it can be resent manually to volunteers or solved in a local computer.

## Methods

### Effect of the configuration parameters

Firstly, we carried out three experiments to evaluate the effects of the configuration parameters on the performance of DENIS. During the period in which the experiments were carried out, the number of volunteer hosts in the network ranged between 17000 and 25000. We launched batches of simulations with the Carro *et al*. model and varied the duration of the simulations, the task deadline, and the size of the simulation batch, as explained in the following subsections.

The three experiments were performed sequentially. Simulation batches were fed consecutively into the system for a total period of 39 days (13 days for each experiment). For each experiment, all the input files were uploaded to the server at once, but only the WUs of the first batch were created and dispatched initially. Then, a server daemon checked the amount of WUs in the dispatch queue every five minutes. When this amount was smaller than 3000, the following batch was automatically added to the queue. This dispatching policy prevented both server overload and work shortage, and also helped to simulate the real behaviour of VC projects, where job batches from different projects may partially overlap in time.

After receiving the results from volunteers, we measured the time necessary to complete each simulation from the moment a WU was created in the system until the corresponding result was marked as valid. Such interval included both the time spent in the server, and the CPU and idle time at the hosts. The idle time for every WU was defined as

$$idle\_time\;\left(\%\right)=\frac{CPU\;time}{completion\;time}*100$$

where *CPU* *time* is the average CPU time of the two redundant tasks of the work unit.

In VC networks, computational throughput may decrease with time due to the tail problem [21]. The completion of the last tasks in the batch may be delayed well after the majority of the jobs have finished, producing what is termed the tail phase of the computation. For each simulation batch, we measured the time elapsed until 80% and 100% of the simulations in the batch were completed (denoted as “100% completion time” and “80% completion time” respectively). in order to characterize the behaviour of the system both before and after the tail phase.

The time distributions resulting from the different experimental conditions were compared with the Wilcoxon rank sum test.

#### Effect of the simulation length

In the first experiment, we launched consecutive batches of 600, 1200, 1800 and 2400-s simulations, repeating the cycle for 13 days. The task deadline was set to 10.5 days and the batch size was set to 10K simulations. In total, 120 batches were fed into the system during the duration of the experiment.

#### Effect of the task deadline

In the second experiment we added batches of 10000 simulations with a length of 1800 s. The task deadline was set from two days to six days in steps of one day for consecutive batches. The cycle was repeated for 13 days, with a total of 141 batches being sent.

#### Effect of the batch size

In the third experiment we consecutively added batches of 10000, 20000, 30000, 40000, 50000 and 60000 simulations. The cycle was repeated during for 13 days, with a total of 73 batches being sent. All simulations were 1800 s long and had a deadline of four days.

### Test case: Population of models using a full-factorial design

When working with populations of models, the generation of a large pool of candidate models is a computational challenge. We tested the performance of DENIS in such situation by replicating the simulations carried out in a recent study [4]. Two populations of models were generated, one with the Shannon *et al*. model [22] and one with the Mahajan *et al*. model [23], by introducing variability into ionic current conductances. For each model, variations were generated using a full factorial approach, that is, considering all possible combinations of values in the range of 0%, ±15%, ±30% values of six model parameters (conductances), which resulted in 15625 combinations. Each combination was stimulated with three different cycle lengths (400 ms, 600 ms and 1000 ms) during 1000 s, which resulted in 93750 simulations in total. In our experiment, we launched all simulations at the same time with a deadline of four days for a first round. Also, in an attempt to accelerate the completion of the job by using the results from the fastest volunteers, we launched the same simulations for a second round, and measured the time spent until the fastest result from the two rounds was validated.

### Comparison with a stand-alone computer

Finally, we launched simulations with the Carro *et al*., Shannon *et al*., and Mahajan *et al*. models in a desktop computer with 4GB of RAM and an Intel Core i5 4-core processor at 2.67GHz, running the stand-alone version of the 64-bit Linux DENIS executable.

## Results

### Effect of the simulation length

**Fig 3:**
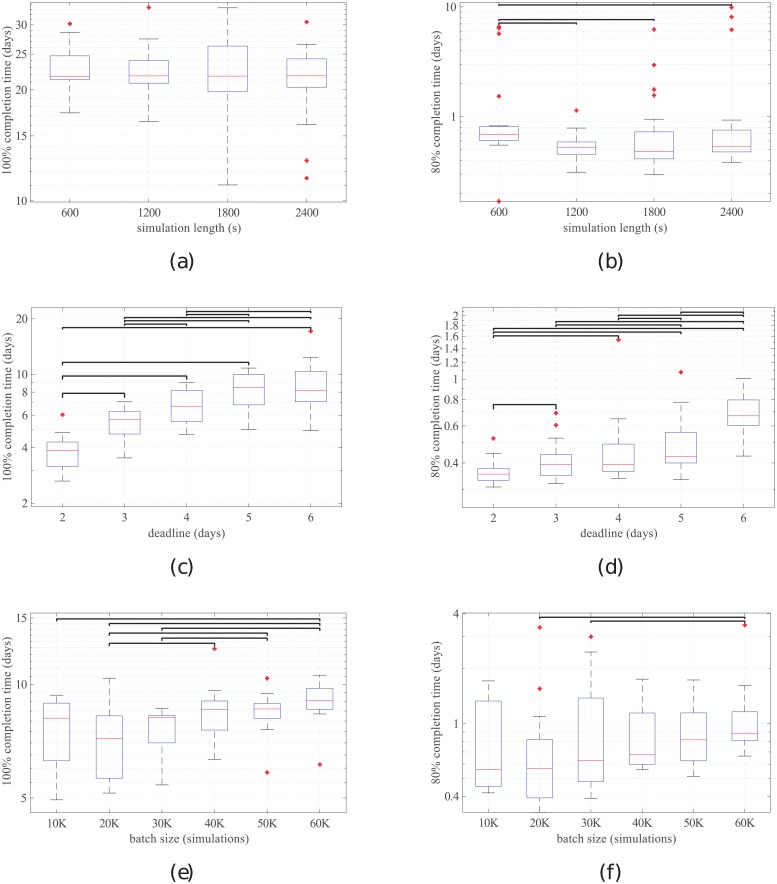
Boxplots of batch completion times for different experimental conditions. (a)–(b): effect of simulation length; (c)–(d): effect of task deadline; (e)–(f): effect of batch size. Black lines indicate significant differences between pairs of boxes (p<0.05).

The time to complete a whole batch of simulations did not significantly increase with the simulations’ length (see Fig 3(a)). The median 100% completion time was between 21.7 and 21.8 days for all lengths. This means that, for any given length, in 50% of the batches there were simulations that took more than 21 days to complete. Since the deadline was 10.5 days, this indicates that in those simulations there was a task which was not completed by two consecutive volunteers, and had to be completed by a third volunteer.

Notably, the median time to complete 80% of the simulations in a batch was significantly higher for 600-s simulations than for the rest (16.6 hours for 600-s simulations *vs*. 12.7, 11.5, and 13.0 hours for 1200-s, 1800-s and 2400-s simulations respectively) (see Fig 3(b)). The median idle times ranged from 96.6 to 94.9% for the different simulation lengths.

In the stand-alone computer, the time to complete a single simulation ranged from 2509.1 s (0.7 hours) for 600-s simulations to 8474.5 s (2.4 hours) for 2400-s simulations. Therefore, if the four cores in the computer were used simultaneously, the time to complete a batch of 10K simulations would range from 72.6 to 245.2 days. Using linear regression, the estimated time to simulate 1 s of the model’s activity was 3.7 s.

### Effect of the task deadline

Of the three studied factors, the task deadline had the strongest impact on completion time. There were significant differences on the 100% completion time for all the deadlines except for the pair of five *vs*. six days (see Fig 3(e)). When looking at 80% completion time, the 6-day deadline group was the slowest to complete, with significant differences with all the rest.

### Effect of the batch size

The median time to complete a batch was significantly higher for larger batches (≥ 40K simulations) than for smaller batches (≤ 30K simulations) in most cases (see Fig 3(c)). However, there were no significant differences among the smaller batches or among the larger batches. In all cases the median completion time was below 10 days. When looking at 80% completion, significant differences only appeared for comparisons with the largest size (60K simulations, see Fig 3(d)).

In the stand-alone computer, the time to complete a single 1800-s simulation was 6694 s. Therefore, if the four cores in the computer were used simultaneously, the time to complete the batches in the experiment would range from 193.7 days for a batch of 10K simulations to 3.2 years for a batch of 60K simulations.

### Test case

**Fig 4:**
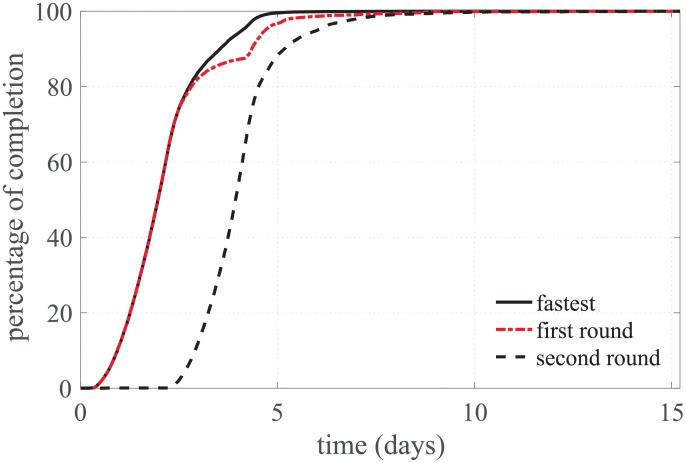
Percentage of completed simulations vs. time for the test case.

The completion times for the test case simulations are depicted in Fig 4 and summarized in Table 1. Launching each simulation twice and waiting just for the fastest result decreased the 100% completion time by almost 41% (first round *vs*. fastest in Table 1). In the stand-alone computer, the time to complete a single simulation was 3004.5 s (0.8 hours) for the Shannon *et al*. model and 1945.9 s (0.5 hours) for the Mahajan *et al*. model. Therefore, if the four cores in the computer were used simultaneously, the time to complete all the simulations in the full-factorial design would be 671.4 days.

**Table 1:** Completion times (in days) for the test case simulations.

	1^*st*^ round	2^*nd*^ round	fastest
80% completion	2.8	4.5	2.8
100% completion	15.1	15.2	9.0

## Discussion

With DENIS, the time to complete a batch of simulations was greatly reduced in comparison with the stand-alone alternative. The 100% completion time was divided by a factor ranging from three to 44, for the shortest (600-s long) and test-case simulations respectively, in comparison with a stand-alone computer. Contrary to stand-alone computers, in which completion time increases proportionally to the length of the simulations, the completion time with DENIS did not significantly increase for longer simulations, which poses a great advantage.

In fact, when considering 80% completion times (Fig 3(b)), the shortest simulations took longer to complete than the rest. This finding, perhaps counter intuitive, may reflect an intrinsic property of volunteer computing systems, which is the coexistence of slow and fast volunteers. Slow volunteers are hosts with tight hardware resources or hosts that allocate less computing time to DENIS jobs. At the beginning of the experiment, all volunteers were idle and waiting for jobs. The first batch to be launched contained 600-s simulations and it was distributed to both fast and slow volunteers. Subsequent batched were distributed to faster volunteers on average, since slow volunteers were still occupied with their first task. Since the time to complete a whole batch is determined by the slowest volunteer, that “faster on average” effect disappeared for 100% completion times. In all experiments, the last 20% simulations in a batch took much longer to complete than the first 80%. For example, 80% of 600-s simulations were completed in 0.7 days, while the remaining 20% took 21.5 days to complete.

In all cases, the computation time in the host was only a small fraction of the total time in the system. In addition to computation time, factors such as the time spent in the dispatch queue or in the *validator*, but specially the idle time in the host added significant delay to the completion time. The main reason behind such a high idle time is that the execution of VC tasks is usually suspended when the computer is in use, in order to be minimally obtrusive for hosts. Also, hosts may be subscribed to other BOINC projects besides DENIS; in such cases the BOINC client schedules jobs belonging to different projects according to several criteria, the most important being the task deadline.

Indeed, the deadline was the factor with the strongest influence in our experiments. Results showed that 80% of simulations tended to be completed well before the deadline, while 100% completion times tended to approach or even surpass twice that limit. In practice, however, task deadlines should not be too tight, since a growing number of volunteers would not be able to complete and report their tasks on time and would not receive credit for their work, which may cause them to abandon the project. Collaborating with a large group of volunteers is essential for the survival of VC projects.

The number of volunteers in a project limits the number of simulations that can be solved simultaneously. Hosts can run multiple DENIS tasks concurrently, so the theoretical upper limit would be determined by the number of available cores. In practice, however, the number of simultaneous simulations will be lower due to the volatile nature of VC resources. Results from the third experiment showed significantly different completion times for batch sizes above and below 30K simulations, which is between one and two times the number of volunteer hosts. In the test case study, with a batch size higher than 90K simulations, the 100% completion time was around 3.75 times higher than the deadline for a single round. According to our results, therefore, DENIS will offer the best performance when the batch size is similar or moderately superior to the number of available hosts.

Results indicate that DENIS is well suited to problems requiring a large number of independent simulations. A clear example of such problems are studies with populations of models like in our test case [4] and others [24–26]. Another example are studies involving model optimization such as [27, 28], where genetic algorithms are used to fit some parameters of the model. The evaluation of each iteration of the genetic algorithm is computationally intensive, but it can be run in parallel. In both kinds of studies, thanks to the small dependence of DENIS with the batch size, the size of the population could be increased without excessively lengthening the completion time.

In such studies, the completion of the tasks could be further accelerated by modifying job dispatching in several ways. One way could be to replicate jobs, like in our test case where each simulation was launched twice, and to wait only for the fastest result; other possibilities include generating more than two tasks per job and using a minimum number of agreeing results for validation [29]. Also, a hybrid approach where simulations that missed the deadline in DENIS were solved in a supercomputer or cluster could be cost-effective, since our results indicate that only a small percentage of tasks would miss a moderate deadline.

Finally, limitations of this work need to be acknowledged. In the first place, changes in the availability of volunteers had an influence in the performance of DENIS. Accounting for this variability is not straight-forward, because not even the number of volunteers correlates perfectly with the amount of work that can be performed in a VC network, since volunteers can individually set quota limits for CPU usage. Given this limitation, we tried to minimize the effect of the variable number of volunteers within each experiment by launching the batches corresponding to the different groups in a cyclic way, without waiting for previous batches to finish execution. This means, for example, that in the first experiment the volunteer network was solving simulations of all sizes at a given point in time. Therefore, the four groups in Fig 3(a) were affected by the variable number of volunteers in a similar way, and it is safe to assume that the differences between groups should not be heavily affected by the volunteer variability. Also, this dispatching policy makes it possible to evaluate the DENIS performance in a scenario as realistic as possible, in which DENIS could be working on different problems simultaneously.

An additional effect of this dispatching policy is the effect of slow *vs*. fast volunteers in short simulations that we discussed previously, and also the delay between the first and second round of simulations in the test case. As a secondary consequence, the groups of simulations in our experiments were not totally independent from each other. Non-independent observations introduce bias and can make a statistical test give too many false positives [30]. In our case, that means that the influence of simulation length, deadline, and batch size could be weaker than our results suggest.

Another limitation regards the expected long-term performance of DENIS. In small-scale VC projects like this one, with work units expected to take a matter of hours to complete and where work is available on a sporadic basis, the biggest challenge is the retention of volunteers, as there is a very high drop-out rate [19]. In our experiments we use a simple First-Come-First-Served job dispatching policy, not taking into account the hosts’ past performance, and the default credit-awarding system [20]. More sophisticated policies could yield better performance [31][16], and improve the retention rate of volunteers [19].

## Conclusion

This paper demonstrates the capabilities of DENIS, a cardiac electrophysiology simulator based on volunteer computing. DENIS greatly outperformed regular stand-alone computers, dividing the time to complete large batches of simulations by a factor ranging from three to 44 in different experiments. Such capacity makes it possible to undertake large cardiac simulation projects without the need for HPC infrastructure.
